# Combination of Atractylenolide I, Atractylenolide III, and Paeoniflorin promotes angiogenesis and improves neurological recovery in a mouse model of ischemic Stroke

**DOI:** 10.1186/s13020-023-00872-z

**Published:** 2024-01-04

**Authors:** Haiyan Li, Wantong Yu, Yong Yang, Sijie Li, Jun Xu, Chen Gao, Wei Zhang, Wenjie Shi, Kunlin Jin, Xunming Ji, Changhong Ren

**Affiliations:** 1 Beijing Key Laboratory of Hypoxia Translational Medicine, Xuanwu Hospital, Center of Stroke, Beijing Institute of Brain Disorder, Capital Medical University, Chang Chun Road 45, Beijing, 100053 China; 2 School of Chinese Medicine, Beijing University of Chines Medicine, Beijing, 100029 China; 3 Department of Pharmacology and Neuroscience, Texas Health Science Center, University of North, Fort Worth, TX 76107 USA

**Keywords:** Cerebral ischemia, Angiogenesis, Nature herbs, Traditional Chinese medicine, *Atractylodes Macrocephala* Koidz, *Paeonia lactiflora* Pallas

## Abstract

**Background:**

Prognosis is critically important in stroke cases, with angiogenesis playing a key role in determining outcomes. This study aimed to investigate the potential protective effects of Atractylenolide I (Atr I), Atractylenolide III (Atr III), and Paeoniflorin (Pae) in promoting angiogenesis following cerebral ischemia.

**Methods:**

The bEnd.3 cell line was used to evaluate the effects of these three compounds on vascular endothelial cell proliferation, migration, and tube formation. Male C57BL/6 mice underwent transient middle cerebral artery occlusion (MCAO), followed by daily intragastric administration of the Chinese medicine compounds to assess their impact on brain protection and angiogenesis. In vivo experiments included measuring infarct size and assessing neurological function. Immunofluorescence staining and an angiogenesis antibody array were used to evaluate angiogenesis in ischemic brain tissue. Functional enrichment analysis was performed to further investigate the pathways involved in the protective effects of the compounds. Molecular docking analysis explored the potential binding affinity of the compounds to insulin-like growth factor 2 (IGF-2), and Western blotting was used to measure levels of angiogenesis-related proteins.

**Results:**

In vitro, the combination of Atr I, Atr III, and Pae enhanced cell proliferation, promoted migration, and stimulated tube formation. In vivo, the combined treatment significantly facilitated neurological function recovery and angiogenesis by day 14. The treatment also increased levels of angiogenesis-related proteins, including IGF-2. Pearson correlation analysis revealed a strong positive association between IGF-2 levels in ischemic brain tissue and angiogenesis, suggesting a good affinity of the compounds for the IGF-2 binding site, as supported by molecular docking analysis.

**Conclusion:**

The administration of Atr I, Atr III, and Pae has shown significant enhancements in long-term stroke recovery in mice, likely due to the promotion of angiogenesis via increased activation of the IGF-2 pathway in ischemic brain tissue.

**Graphical Abstract:**

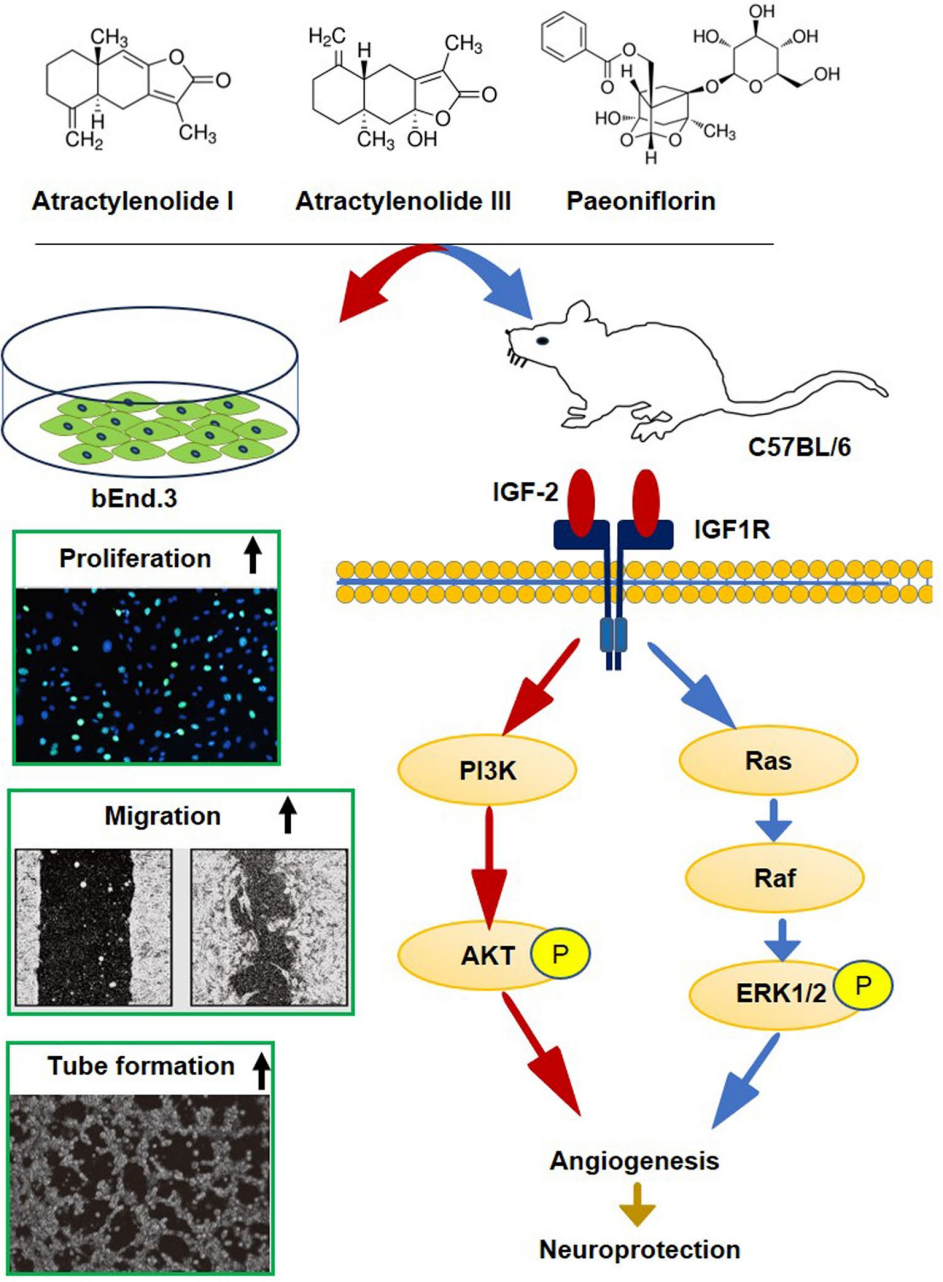

## Introduction

Ischemic stroke, a leading cause of both mortality and disability, places a significant burden on the elderly population and its global incidence continues to rise [1]. Despite the availability of intravenous thrombolysis and arterial thrombectomy in reducing neuronal damage in ischemic stroke, their utilization is limited to a small percentage of patients. Up to 95% of stroke patients are unable to receive these treatments due to time constraints and contraindications [2–4]. Therefore, the focus on post-stroke recovery remains crucial, regardless of the availability of thrombectomy and thrombolytic therapy [5, 6]. There is an urgent need to prioritize initiatives that promote neurological recovery and improve prognosis following ischemic stroke [7].

Recovery of neurological function after an ischemic stroke is closely associated with various factors, including angiogenesis, neurogenesis, and synaptic plasticity [8]., Angiogenesis is particularly crucial when cerebral blood flow obstruction, as it enhances the oxygen and nutrient supply to the affected brain tissue, preventing further damage [9]. Studies show that stroke patients with a higher density of new blood vessels around the damaged area have better survival rates [10]. Animal experiments have indicated that drugs inhibiting blood vessel cell activity can impede angiogenesis and result in poor neurological recovery post-stroke [9]. Therefore, angiogenesis is a key mechanism for repairing damaged brain tissue after ischemic injury.

Traditional Chinese Medicine (TCM), with its rich history and unique theoretical framework, has been widely recognized for its therapeutic advantages in treating various diseases, including ischemic stroke [11–14]. TCM theory suggests that ischemic stroke primarily results from blood stasis, qi deficiency and stagnation [15]. The core syndromes include blood stasis syndrome, phlegm, Yin deficiency, Qi deficiency, and hyperactivity of liver Yang among others [16]. Ischemic stroke is considered to occur in the brain and closely linked to the heart, liver, spleen, and kidneys. Its pathogenesis involves underlying deficiency and apparent excess, imbalance of yin and yang, and disorder of qi and blood circulation [16]. Modern clinical TCM research has identified the five major pathogenic factors of stroke as wind, fire, phlegm, stasis, and deficiency [17]. Stroke treatment in TCM employ methods such as dispelling wind, clearing fire, resolving phlegm, promoting blood circulation, alleviating water retention and nourishing the liver and kidneys are used [17].

*Atractylodes macrocephala* Koidz (AMK) and *Paeonia lactiflora* Pallas (PLP) are two herbs commonly used in TCM prescriptions [18, 19]. AMK is known to target the spleen and stomach channels, thereby tonifying spleen, replenishing ‘Qi’ (vital energy), removing dampness, and alleviating water retention [20]. PLP is associated with the liver channels and is known for calming liver-yang, nourishing blood, and promoting blood circulation [21]. As a result, formulas containing AMK and PLP are utilized in stroke treatment due to their combined effects of strengthening the spleen and reducing water retention, as well as calming liver Yang [22, 23].

Contemporary research in TCM has pinpointed several potent extracts that possess neuroprotective qualities akin to those found in TCM prescriptions [24]. Atractylenolide I (Atr I) and Atractylenolide III (Atr III), active constituents of AMK, have shown a range of benefits in pharmacological studies, including neuroprotection, anti-neuroinflammatory properties, antioxidant effects, anti-allergic effects, anti-cancer properties, and cognitive enhancement [18, 25, 26]. Specifically, in a mouse model of middle cerebral artery occlusion (MCAO), Atr III was found to reduce the size of brain infarct, restore cerebral blood flow, lessen brain swelling, and improve neurological outcomes [27]. Network pharmacological analysis have pinpointed Atr I as a crucial active component of AMK in fight against ischemic stroke [28]. Similarly, network pharmacology research indicates that Paeoniflorin (Pae), an active component of PLP, along with atractylenolide, plays a crucial role in ischemic stroke [28, 29]. Preclinical meta-analysis has demonstrated that Pae exhibits neuroprotection against cerebral ischemia by modulating a variety of biological processes, including the protection of brain-blood barrier protection, modulation of neuroinflammation, reduction of oxidative stress, and inhibition apoptosis [30]. In a rat model of stroke, Pae was observed to increase the number of endothelial progenitor cells and promote the release of pro-angiogenic factors [31]. Additionally, recent studies have revealed that Pae not lonely rescues neuroinflammation but also promotes neurogenesis, leading to enhanced functional recovery in MCAO rats [32]. Drawing from the TCM principle of compatibility, this study aims to explore the innovative hypothesis that administering a combination of Atr I, Atr III, and Pae can promote angiogenesis in the brain following a stroke, thereby improving sustained functional recovery after ischemic stroke.

## Materials and methods

### Chemicals and reagents

Antibodies: BrdU (11,170,376,001) was purchased from Roche, CD31 (222,783) was purchased from Abcam. Alexa Fluor 488-conjugated goat anti-rabbit IgG (R37116), and Alexa Fluor 594-conjugated donkey anti-mouse IgG (R37115) were purchased from Invitrogen. Phosphatidylinositol-3-hydroxykinase (PI3K, 17,366), phosphorylated protein kinase B (PKB; also known as AKT) (p-AKT, 4060), Rat sarcoma (Ras, 67,648), phosphorylated rapidly accelerated fibrosarcoma (p-Raf, 20,011), and phosphorylated extracellular signal-regulated kinase (p-ERK1/2, 4370) were purchased from Cell Signaling. β-actin (A5441) was purchased from Sigma-Aldrich.

Reagents: Dulbecco’s Modified Eagle Medium (DMEM, 1966-025) and fetal bovine serum (FBS, 10,099,141) were purchased from Gibco, penicillin-streptomycin (15,140,122) was purchased from Thermo Fisher. Atr I (A2737), Atr III (A2987), and Pae (P0038) were purchased from Sigma. Cell counting kit-8 (CCK8) assay (WST-8) was purchased from Dojindi Labs. matrigel (356,234) was purchased from Corning. FITC-conjugated Lycopersicon esculentum Lectin (ZB0112) was purchased from Vector.

### Cell culture

bEnd.3 cells (ATCC, CRL-2299) were cultured in complete DMEM, supplemented with 10% (v/v) FBS, and 1% (v/v) penicillin-streptomycin. Cells were seeded at a density of 4 × 10^4^ cells / cm^2^ in dishes, with the medium refreshed three times weekly.

Oxygen–Glucose Deprivation (OGD) / Reoxygenation: To simulate ischemia/reperfusion injuries in vivo, the culture medium was replaced with FBS - glucose - free DMEM, and the plates were then placed in an anaerobic chamber with an atmosphere 95% N_2_ and 5% CO_2_, and incubated for 4 h. Following OGD, the culture medium was replaced with complete DMEM, and the cells were moved to a normoxic incubator. Atr I, Atr III, and Pae were initially dissolved in pure dimethyl sulfoxide (DMSO) and subsequently diluted to the desired concentrations using DMSO.

### CCK8 assay

Cell viability was assessed using the CCK8 assay. bEnd.3 cells were seeded into 96-well plates with compounds at a density of 5 × 10^3^ cells/well. After 24 h, cells underwent 4 h of OGD followed by 4 h of reoxygenation, with the compounds present in the medium throughout both periods. Post-treatment, the medium was replaced with a mixture of medium and CCK8 solution at a ratio of 10:1, and the wells were incubated for 1 h at 37℃. Absorbance (OD) at 450 nm was evaluated using a Multiscan MS spectrophotometer (THERMO, JS-THERMO Varioskan Flash, American). n = 4 per group.

### Wound healing assay (horizontal migration) and transwell assay (vertical migration)

The wound healing assay was carried out using ibidi culture-insert 2 well (Ibidi, 80,206) to assess horizontal cell migration. A culture-insert was placed into a 35 mm culture dish, and 50 µL cell suspension (4 × 10^5^ cells/ml) was added into each well. After the cells reached confluence, the insert was removed, and the dish was filled with either complete DMEM or FBS-free DMEM containing compounds. After 24 h, cell migration was captured using an Olympus TH4-200 microscope, and the migration area was quantified with Image J software. The ratio of migrated area to scratch area was then calculated.

For the transwell assay, which measures vertical migration of bEnd.3 cells, 500 µL of complete medium was added to the lower chamber. In the upper chambers (Corning, 8.0 μm), 200 µL of a cell suspension (7.5 × 10^4^ cells/ml) was seeded without FBS in the presence or absence of the test compounds. After 18 h, cells that had migrated to the lower surface of the membrane were stained with 0.1% crystal violet. Cells that had not migrated and remained on the upper surface were removed with a cotton swab. The number of migrating cells was counted in five random fields for each sample.

### Tube formation assay

The tube formation assay was performed to evaluate the morphogenesis and tube formation capacity of the bEnd.3 cell. A 96 well plate (ibidi, 89,646) was coated with 10 µL matrigel, and allowed to solidify at 37 ℃ for 30 min. bEnd.3 cells with or without compounds, were seeded onto the Matrigel-coated plates at a density of 2 × 10^4^ cells per well, and incubated at 37 ℃ for 2 h. Tube formation was observed and quantified using an inverted light microscope (OLYMPUS, TH4-200), with analysis conducted across five independent fields.

### Animals model and drug administration

Male C57BL/6 mice weighing 22-25 g were obtained from Vital River Laboratory Animal Technology Co. Ltd (SYXK(Jing)2022-0052). All animal experiments were approved by the Institutional Animal Care and Use Committee of Capital Medical University (Approval no. AEEI-2021-255) and carried out in a facility with a 12-hour light/dark cycle. Anesthesia was induced using 1.5% enflurane, 30% O_2_, and 68.5% N_2_O. The middle cerebral artery occlusion (MCAO) model was performed as previously described [33], with a laser speckle contrast imager (PSI system, Perimed Inc.) confirming proper placement of the filament in the right middle cerebral artery. A temperature-controlling pad (Harvard Apparatus, MA, USA) was used to maintain the mice’s body temperature during surgery (Harvard Apparatus, MA, USA). Immediately post-surgery, buprenorphine SR (1 mg/kg) was administered intraperitoneally for analgesia. The mice were randomly divided into sham-operated, MCAO, and MCAO + Compounds (Atr I + Atr III + Pae) groups. All mice received intraperitoneal injections of 5-bromo2-deoxy-uridine (BrdU, 50 mg/kg) twice daily for 14 days, starting the day after MCAO. This regimen included an initial injection 30 min post-MCAO surgery, MCAO surgery, followed by twice-daily injections for the subsequent 13 days.

Atr I, Atr III, and Pae were dissolved in normal saline to create a suspension, with each drug at a concentration of 2 mg/mL. The MCAO + Compounds group received a mixture of these compounds (20 mg/kg, 0.2 mL) by intragastric administration at 30 min after reperfusion daily until the mice were sacrificed. The Sham group and MCAO group received an equivalent volume of vehicle solution.

Experiment 1: To determine the most effective dosage, the compounds were administered daily in three different doses: 5, 10, and 20 mg/kg/day, or saline. These dosage levels were chosen based on ranges reported in the literature for similar compounds tested in vivo in mice [26, 34, 35]. The mice were sacrificed 3 days after MCAO. In this initial experiment, twenty mice were randomly assigned into four groups: MCAO + Vehicle (n = 5), MCAO + 5 mg/kg/day (n = 5), MCAO + 10 mg/kg/day (n = 5) or MCAO + 20 mg/kg/day (n = 5).

Experiment 2: To investigate the effects of compounds on post-stroke angiogenesis, the compounds was administered once daily once daily until sacrifice. The dosage was given according to the optimal concentration selected from experiment 1. In the second experiment, forty-nine mice were randomly assigned into 6 groups: Sham 7d (n = 8), MCAO 7d (n = 8), MCAO + Compounds 7d (n = 8), Sham 14d (n = 5), MCAO 14d (n = 10), MCAO + Compounds 14d (n = 10).

### Laser speckle imaging

Laser speckle imaging was used to monitor regional cerebral blood flow (CBF) before and after ischemia (10 min after MCAO). The model was deemed successful if the CBF on the side of infarcted decreased to less than 20% of the contralateral side [36].

### Infarct size measurement

The size of Infarct was assessed using a 2% solution of 2,3,4-triphenytetrazolium-chloride 72 h after MCAO, in accordance with the method previously described [37].

### Neurological function assessment

Neurological function was assessed using the Rota-rod test and the Beam walking test. Mice underwent behavioral training for three days before surgery. During the Rota-rod test, each mouse was placed on a rod that progressively accelerated from 4 rpm to 40 rpm over 5 min. The time the mouse remained on the rod, up to a maximum of 300 s, was recorded. For the Beam walking test, mice were trained to traverse at 6 mm wide, 100 cm long beam. and the number of foot faults was recorded. Performance was scored on a 6-point neurological scale, with higher scores indicating superior neurological function. These experiments and the subsequent data analysis were conducted in a double-blind manner.

### Immunofluorescence staining

Mouse brains were harvested following cardiac perfusion with 0.9% saline and 4% paraformaldehyde. Immunofluorescence staining was carried out as previously described [38], using primary antibodies: mouse monoclonal anti-BrdU (1:500) and rabbit monoclonal anti-CD31 (1:500). Secondary antibodies included Alexa Fluor 488-conjugated goat anti-rabbit IgG (1:300) and Alexa Fluor 594-conjugated donkey anti-mouse IgG (1:300). Vessels were stained with FITC-conjugated Lycopersicon esculentum Lectin (1:300). For BrdU staining, brain slices were treated with 2 M HCl at 37 °C for 20 min, followed by incubation with 1% BSA, and then rinsed with 0.1 M borate buffer (pH 8.5) for 10 min.

For bEnd.3 cells: Coverslips were placed in a 24-well plate, and cells were seeded at a density of 5 × 10^4 cells per well. After 24 h, cells underwent 4 h of oxygen-glucose deprivation (OGD). Then, the culture medium was replaced with complete DMEM containing 30 µM BrdU. Four hours post-treatment, immunofluorescence staining was performed using the primary antibody mouse monoclonal anti-BrdU (1:500) and the secondary antibody Alexa Fluor 488-conjugated donkey anti-mouse IgG (1:300).

### Angiogenesis antibody array

To assess the differentially expressed proteins (DEPs) at day 7 after MCAO, we utilized the RayBio® Mouse Angiogenesis Antibody Array G Series 1 from RayBiotech, Inc., USA. This array detects 24 mouse angiogenic factors and was conducted according to the manufacturer’s instructions. Briefly, we added 100 µL of protein sample to each well and subsequently added biotinylated antibodies and a fluorescent streptavidin agent. Each protein was represented by duplicate dots on the array, which were scanned using an InnoScan 300 Microarray Scanner (Innopsys, France) at a 532 nm wavelength and 10 μm resolution. We considered proteins with an expression fold change greater than 2 or less than − 2 and an adjusted P-value below 0.05 to be significantly differentially expressed. Data analysis was performed using RayBiotech’s Quantibody® Q-Analyzer software.

### Functional enrichment analysis

We annotated protein function using Gene Ontology (GO) analysis, which includes: biological process (BP), molecular function (MF), and cellular component (CC). We mapped all DEPs to GO database terms and identified significantly enriched GO terms among the DEPs using a p-value threshold of less than 0.05.

Additionally, we performed Kyoto Encyclopedia of Genes and Genomes (KEGG) pathway analysis to link genomic information with higher order functional information. All DEPs were mapped to the KEGG database to identify significantly enriched pathways, again using a p-value threshold of less than 0.05.

### Molecular docking

We used AutoDock 4.2 software with the Lamarckian genetic algorithm to determine the optimal binding positions between the ligands (Atr I, Atr III, and Pae) and the IGF-2 protein receptor (UniProtKB AC/ID: 5L3M) [39]. The Lamarckian genetic algorithm (LGA) was utilized to determine the optimal binding position between ligand and receptor. The Atr I, III, and Pae Atr compound molecular files were obtained from PubChem (https://pubchem.ncbi.nlm.nih.gov/) [40]. We set the grid box coordinates and sizes based on the target proteins, included non-polar hydrogen, and used the compound molecules as ligands. The binding conformation with the lowest free binding energy was selected for output results, which were then visualized using PyMol software.

### Western blot

Brain tissue from the infarct hemisphere was harvested on day 7 post-MCAO, weighed, and lysed in RIPA buffer using an ultrasonicator. The supernatant was collected, and protein concentration was determined using the BCA assay. We loaded 30 µg of protein per lane for separation by SDS-PAGE and subsequent transfer onto PVDF membranes. The membranes were blocked at room temperature for 1 h and then incubated overnight at 4 °C with primary antibodies against PI3K, p-AKT, Ras, p-Raf, and p-ERK1/2 (all at 1:1000 dilution). The target proteins were visualized using a chemiluminescent substrate from GE Healthcare, UK, with β-actin serving as the loading control (1:3000). Protein band densities were quantified using ImageJ software (NIH, Bethesda, MD, USA).

### Statistical analysis

Data are presented as mean ± SD. Statistical analysis was performed using SPSS for Windows (version 19.0; SPSS, Inc., New York, NY, USA). The GO and KEGG pathways analysis were performed by R packages named ggplot2. The differences between the two groups were assessed by Student’s t-test, Groups greater or equal to 3 were assessed using one-way ANOVA. Data for neurobehavioral tests were analyzed by two-way repeated measures (RM) analysis of variance (ANOVA). Pearson correlation analysis was used to analyze the relationship between histological parameters and neurological behaviors. Fisher’s exact test was used to test the significance of GO terms and KEGG pathway identifier enrichment in the differentially expressed protein/gene list. A p-value of less than 0.05 was considered significant.

## Results

### Combination of compounds promotes cell proliferation in vitro

**Fig. 1:**
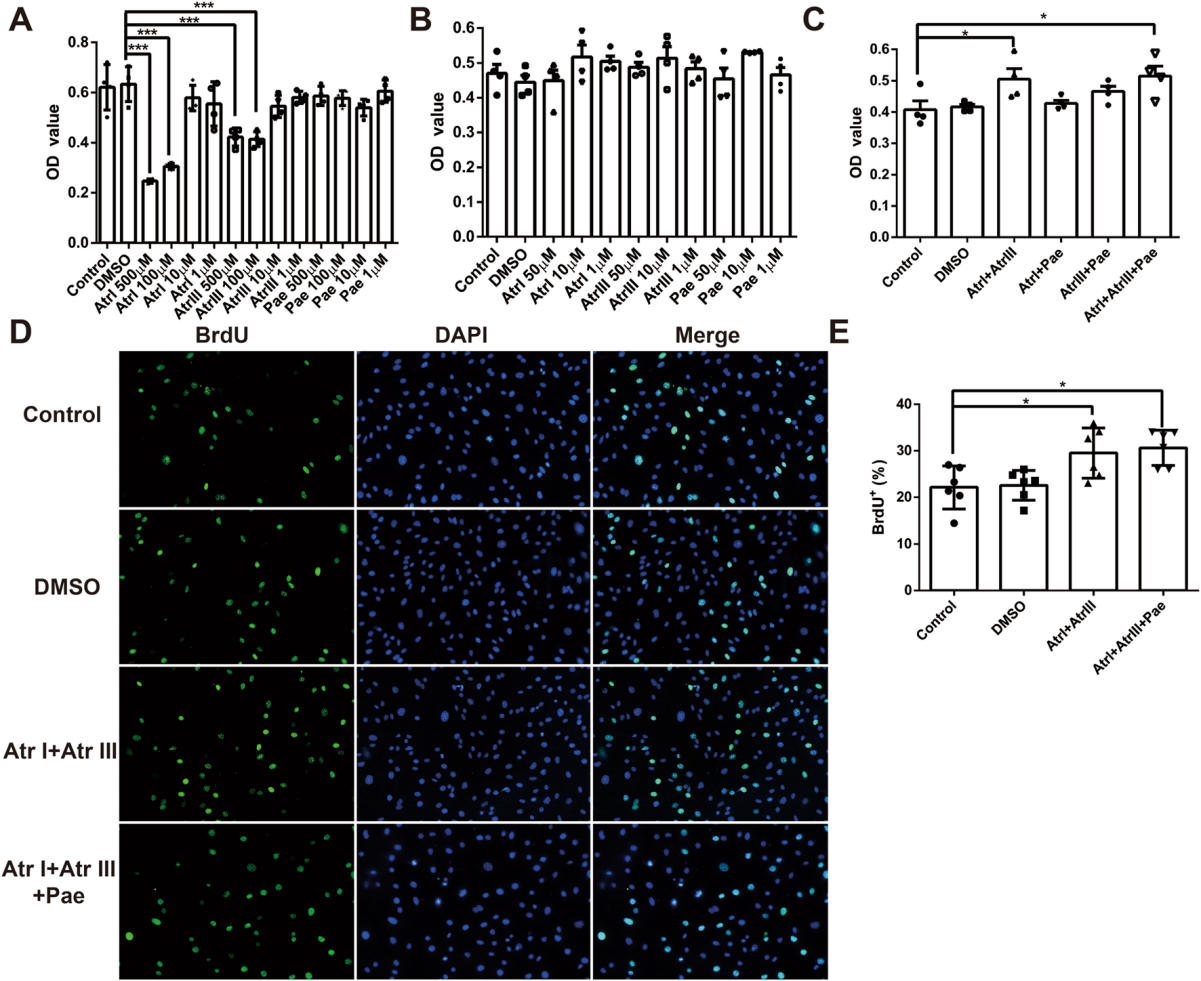
Combination of compounds promotes cell proliferation in vitro. **A** CCK8 assay was performed to evaluate the different doses of Atr I, Atr III, and Pae on cell survival. The result demonstrates the toxicity at high concentrations of the compounds. **B** CCK8 assay was performed to evaluate the effects of Atr I, Atr III, and Pae alone within the non-toxic range, illustrates the efficacy of each compound when used individually. **C** CCK8 assay was performed to assess the synergistic effects of combining Atr I, Atr III, and Pae on cell proliferation. **D** Images represent immunofluorescence staining of BrdU. **E** Bar graph shows the percentage of BrdU positive cells. Data are expressed as mean ± SD. *n* = 4 per group. *p < 0.05, ***p < 0.001 vs. Control group

The CCK-8 assay was used to evaluate the cytotoxicity of the compounds and their effect on cell proliferation. The results showed that Atr I was non-toxic to normal cells in the range of less than 50 µM and did not affect cell proliferation (Fig. 1A and B). Similarly, the safe dose of Atr III is less than 50 µM, and the safe dose of Pae is less than 500 µM (Fig. 1A and B). Since compounds of different concentrations alone could not promote cell proliferation (Fig. 1B), we combined these compounds in pairs to explore their effects on proliferation. CCK-8 results showed that the combination of 10 µM Atr I and 10 µM Atr III and the combination of Atr I, Atr III and Pae compounds promoted proliferation compared with the control group (Fig. 1C). To further confirm the promoting effect of Atr I combined with Atr III on cell proliferation, BrdU staining was performed. The results showed that Atr I combined with Atr III remarkably increased the rate of BrdU^+^ cells (Fig. 1D and E), suggesting that Atr I combined with Atr III significantly promotes the proliferation of bEnd.3 cells.

## Combination of compounds promotes cell migration in vitro

**Fig. 2:**
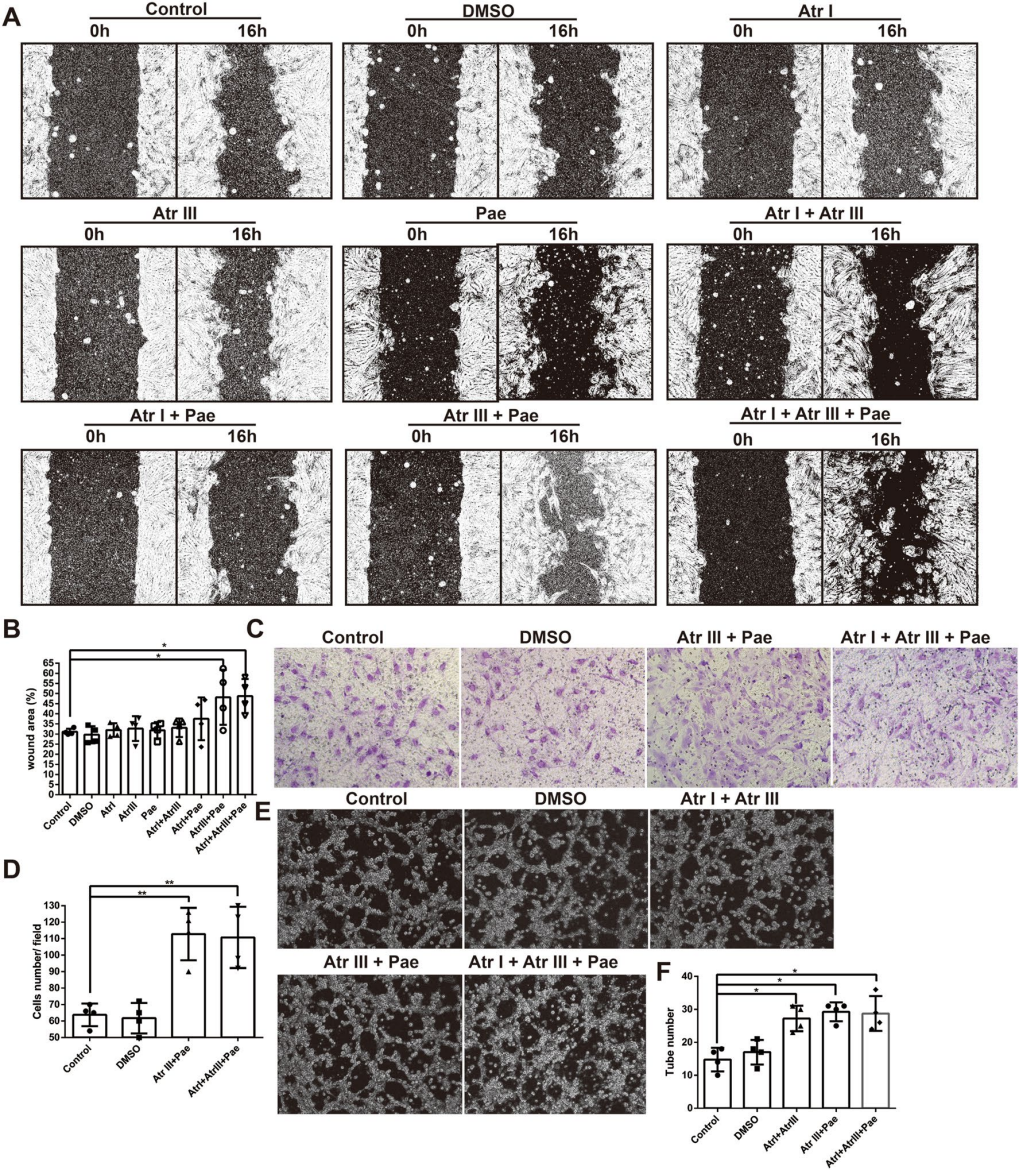
Combination of compounds promotes cell migration and tube formation in vitro. **A** Representative images of cell horizontal migration following treatment with Atr I, Atr III, and Pae, both individually and in combination. **B** Bar graph depicting the quantification of cell horizontal migration (the ratio of migrated area to scratch area). **C** Representative images of cell transwell migration assays treated with the combination of Atr III and Pae. **D** Bar graph shows the quantification of cell number. **E** Representative images of cell tube formation results. **F** Bar graph depicting the quantification of tube number. Data are expressed as mean ± SD. *n* = 4 per group. *p < 0.05, **p < 0.01 vs. Control group

We investigated the effects of compounds on the horizontal and vertical migration of bEnd.3. The results of horizontal migration showed that compound alone did not promote cell migration. Atr III combined with Pae significantly promoted cell horizontal migration at 16 h, and the combination of Atr I, Atr III, and Pae compounds did not further promote cell migration (Fig. 2A and B). And in the transwell assay (vertical migration), Atr III combined with Pae played the same role (Fig. 2C and D).

### Combination of compounds promotes tube formation in vitro

Based on the results of cell proliferation and cell migration, we tested the effect of Atr III combined with Atr I and Atr III combined with Pae on angiogenesis using a tube-formation assay The results showed that, Atr III combined with Atr I and Atr III combined with Pae promoted cell tube formation, while the combination of Atr I, Atr III, and Pae compounds did not further promote cell tube formation (Fig. 2E and F).

### The combination of atr I, Atr III and Pae promotes neurological function recovery

Based on the above in vitro results, we observed that the combined application of the compound was more effective than their individual application. Therefore, we proceeded to explore the effect of the combined application of the three compounds on brain protection and angiogenesis in the following in vivo experiments.

**Fig. 3:**
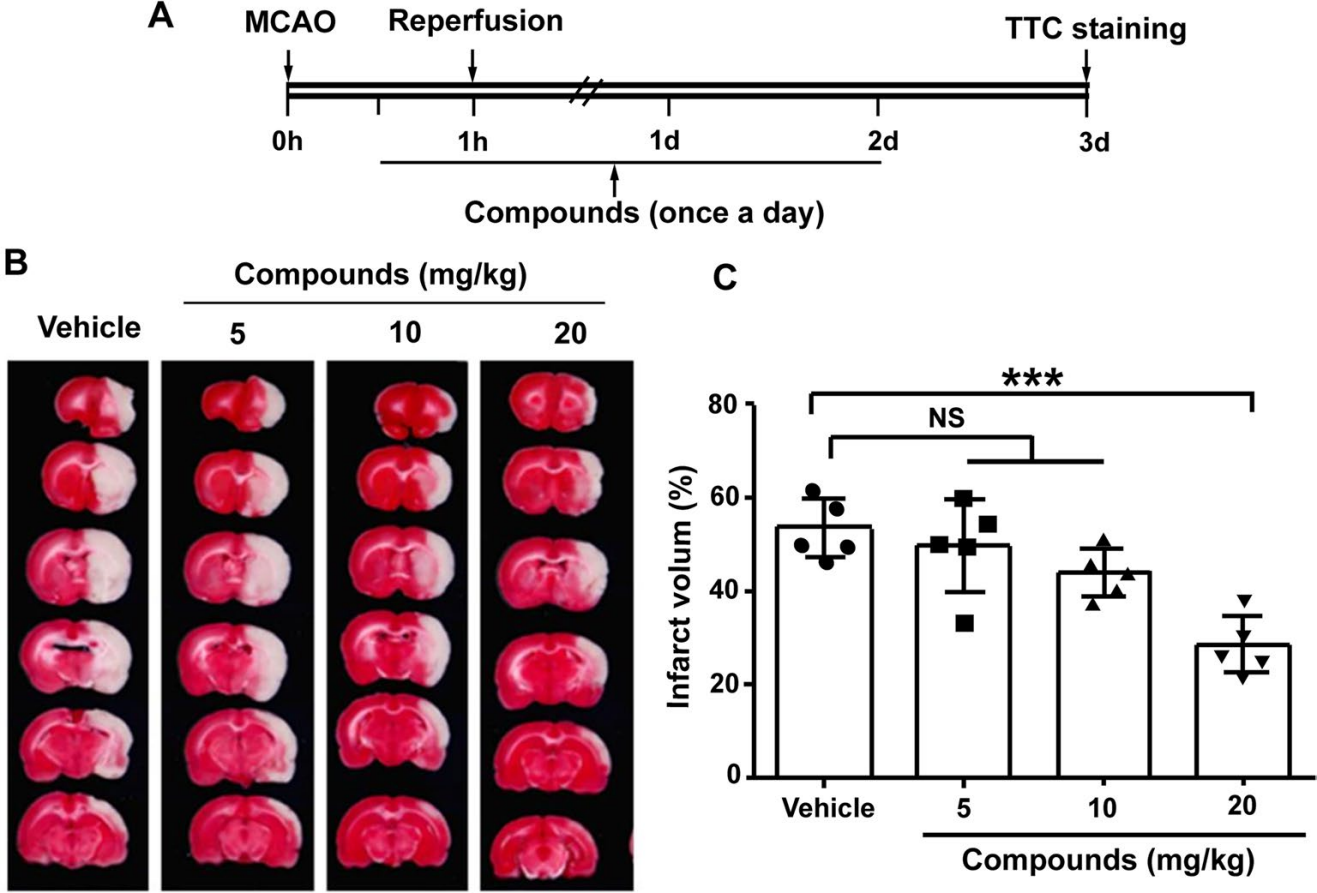
The neuroprotective effect of compounds on MCAO in mice. **A** Timeline for determining the optimal post-stroke dosage in mice, with compounds administered daily via intragastric gavage at doses of 5, 10, and 20 mg/kg/day (or saline). **B** Treatment with compounds led to a decrease in infarct volume. Shown are representative brain slices with triphenyltetrazolium chloride (TTC)-stained infarcts from each group at 3 days post-MCAO. **C** Quantification of infarct volume at 3 days after MCAO. *n* = 5 per group. ***P < 0.001, vs. vehicle group

The compounds were administered according to the experimental setup shown in Fig. 3A. After cerebral ischemia, the infarct volume was significantly reduced at 72 h with incremental doses of compounds at 5, 10, and 20 mg/kg (P > 0.05, P > 0.05, and P < 0.001, respectively; Fig. 3B and C). Notably, the beneficial effects were observed at doses of 20 mg/kg. Therefore, a dose of 20 mg/kg was selected for subsequent experiments to investigate its role.

**Fig. 4:**
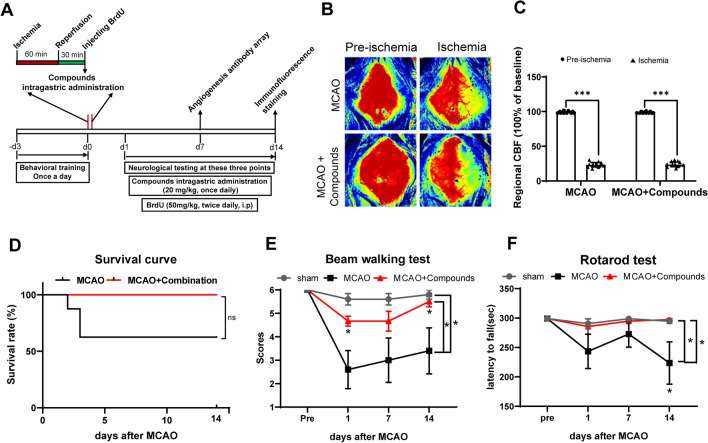
Combination of Atr I, Atr III, and Pae applied after MCAO reduces mortality and neurological dysfunction. **A** Experimental design schematic. **B** Representative laser speckle imaging. **C** Histogram of cerebral blood flow. P < 0.001, N = 10 per group. The result showed no difference in the rate of decline in cerebral blood flow between the two groups. **D** Survival curve up to 14 days after MCAO. (E, F) Neurological behavior tests were performed with **E** Beam walking test and **F** Rotarod test. *n* = 5 mice for MCAO group, *n* = 8 for MCAO + Compounds group. *p < 0.05 vs. MCAO control group

The overall experimental procedure was conducted in accordance with Fig. 4A. Figure 4A and B indicated no difference in the rate of decline in cerebral blood flow between the MCAO group and MCAO + compounds group. Brain protection in MCAO model mice was assessed by calculating the mortality rate and neurological function evaluation of the mice. The results showed that the mortality of mice in the MCAO + Compounds group was significantly lower than that of mice in the MCAO group (Fig. 4D). Neurological function tests were performed on 1, 7, and 14 days after surgery. In the Beam walking test, treatment with compounds exhibited better performance as revealed by significantly increased scores following ischemic stroke (Fig. 4E). In the Rota-rod test, the mice in the MCAO + Compounds group stayed on the rotating rod for a longer time than those in the MCAO group (Fig. 4F). These neurobehavioral test outcomes suggest that the combination of the three compounds can promote the recovery of neurological function in MCAO mice.

## The combination of atr I, Atr III and Pae promotes angiogenesis

**Fig. 5:**
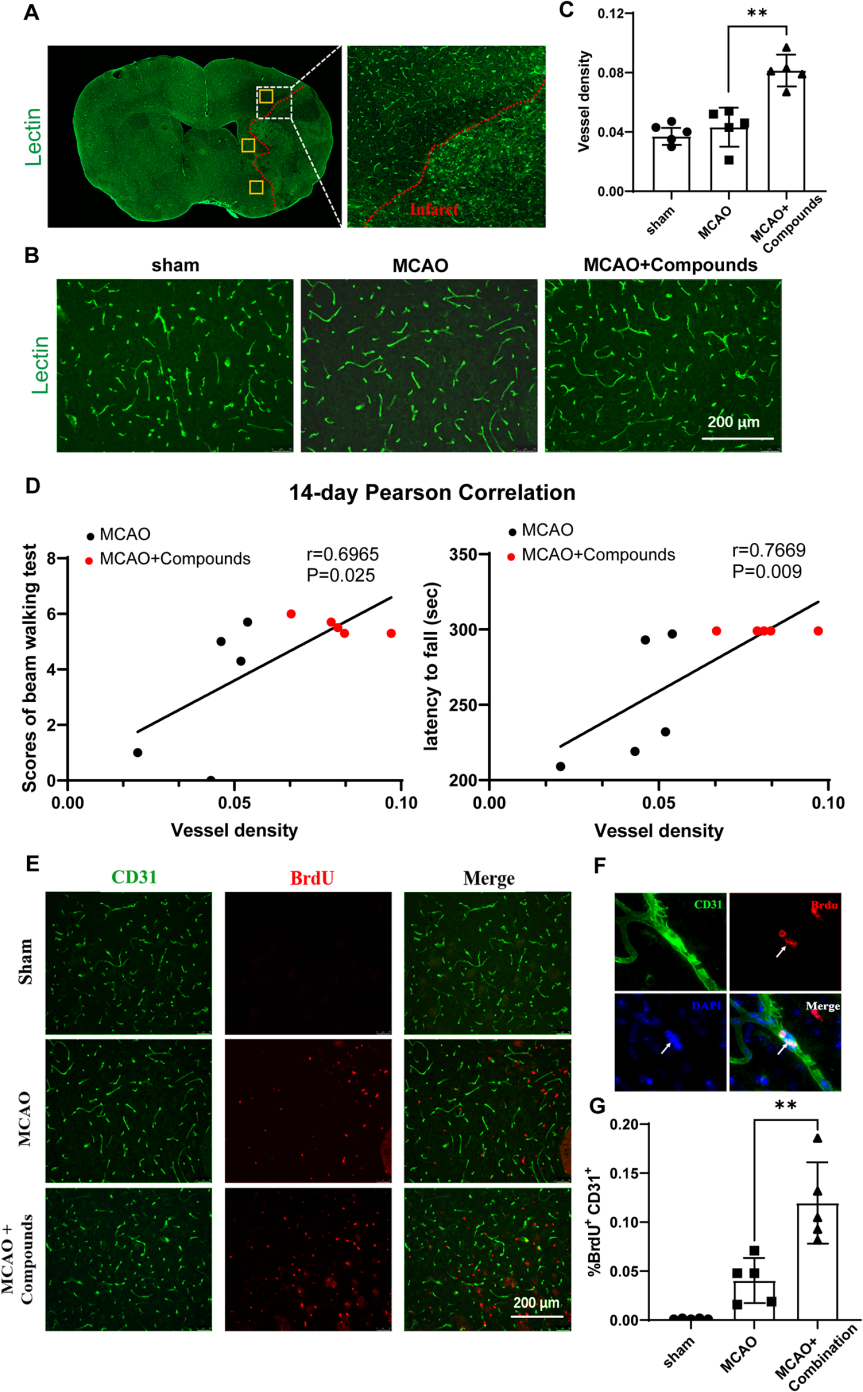
Combination of Atr I, Atr III, and Pae promotes neurological function recovery and promotes angiogenesis in peri-infarct area after MCAO. **A** Representative image of Lectin fluorescence in a coronal brain section at 14 days after MCAO. *Yellow boxes* show the peri-infarct areas where images from **B** were taken. **B** Representative images showing Lectin fluorescence in the ipsilateral peri-infarct area. **C** Quantification of the vessel density in the peri-infarct area, the vessel density for each selected region was measured by ImageJ software (National Institutes of Health, MD, USA) using the following equation [area of vessel (Lectin positive)/total area of the selected region] ×100%. **D** Pearson correlation of vessel density and scores of Beam walking test (left panel) or the latency to fall off the rotarod (right panel) on day 14 post-MCAO. **E** Representative images of CD31 (green) and BrdU (red) immunofluorescence in the peri-infarct area in each group at 14 days after MCAO. **F** Double immunostaining CD31 (green), BrdU (red) and DAPI (blue). **G** Quantification of the number of BrdU + and CD31 + cells. *n* = 5 mice for each group. **p < 0.01 vs. MCAO control group

To determine whether these compounds could promote angiogenesis, Lectin was used to stain vascular endothelial cell, and vessel density in the peri-infarction was calculated (Fig. 5A). Treatment with compounds significantly increased vessel density in the peri-infarct area compared to the MCAO mice (Fig. 5B). To further address whether vessel density was associated with the improvement of neurological function, we performed a Pearson correlation analysis and found a strongly positive correlation between vessel density and performance in the Beam walking test (14 days after MCAO) and Rota-rod test (14 days after MCAO) (Fig. 5C).

To further assess the impact of the combination of three compounds on angiogenesis, we quantified the number of new vascular endothelial cell in the peri- infarct region. CD31 and BrdU immunostaining were performed on coronal brain Sect. 14 days after MCAO. The findings indicated that the number of BrdU ^+^/CD31 ^+^ cells in the peri- infarct region was significantly higher in the MCAO + Compounds group than that in the MCAO group (Fig. 5). The results revealed that the combination of Atr I, Atr III, and Pae promotes angiogenesis in the peri-infarct area of infarction after MCAO.

### The combination of atr I, Atr III and Pae promotes angiogenesis through IGF-2

**Fig. 6:**
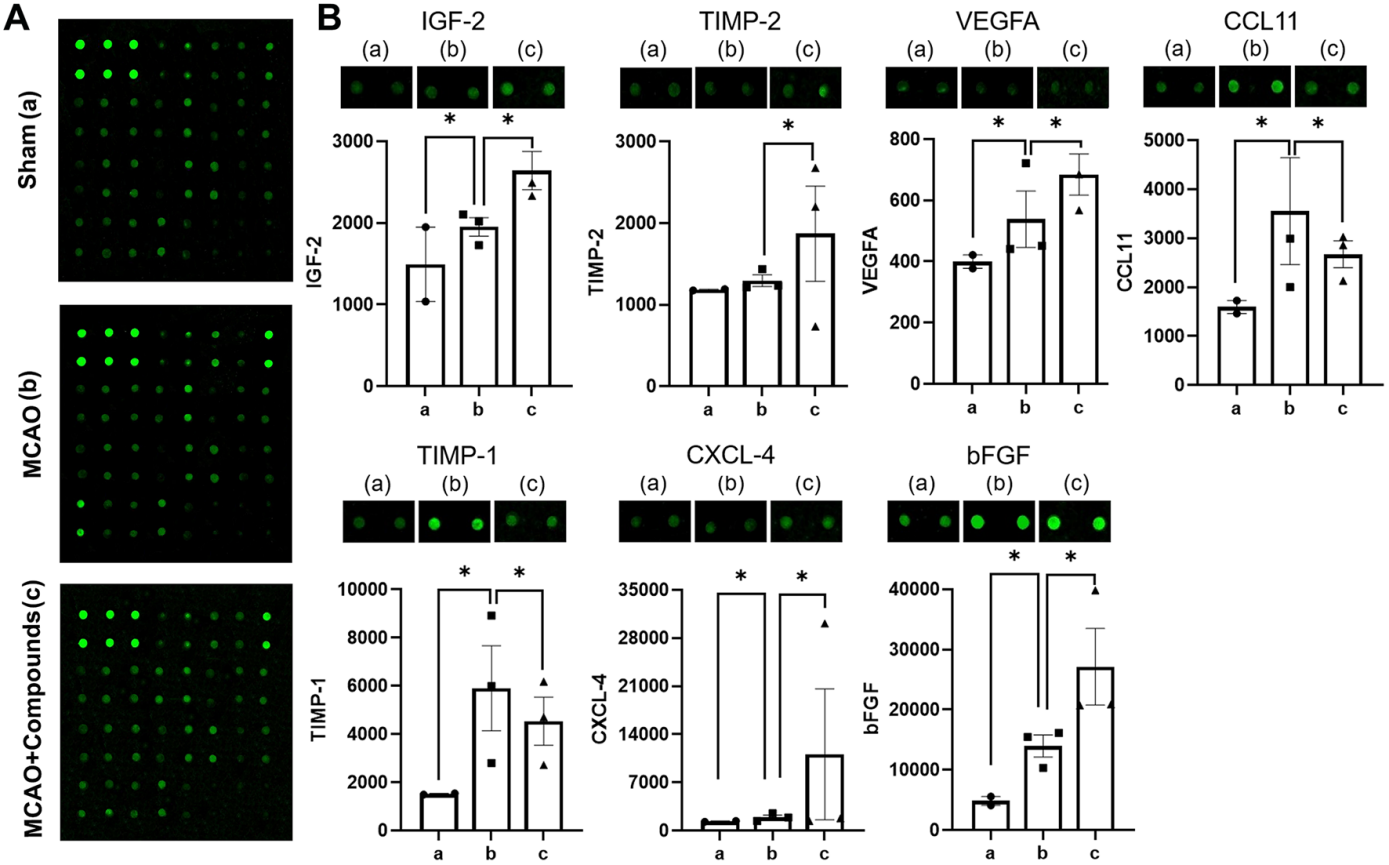
Differentially expressed proteins between MCAO group and MCAO + compounds group. ^*^log FC > 0.263 vs. Sham group, ^*^log FC > 0.263 vs. MCAO control group

To further investigate the molecular mechanism by which these compounds enhance angiogenesis, we compared the expression levels of angiogenesis-related proteins between the MCAO group and the MCAO + compounds group using an Angiogenesis Antibody Array. Among the 23 target proteins, seven proteins showed significant difference in expression between the two groups (Fig. 6). In the MCAO + Compounds group, the expression of insulin growth factor 2 (IGF-2), Metalloproteinase inhibitor 2 (TIMP-2), Vascular endothelial growth factor A (VEGFA), C-X-C motif chemokine 4 (CXCL-4), and Fibroblast growth factor (bFGF) were significantly increased compared to the MCAO group. On the other hand, the expression of C-C motif chemokine 11 (CCL11) and Metalloproteinase inhibitor 1 (TIMP-1) were significantly reduced in the MCAO + Compounds group as opposed to the MCAO group.

**Fig. 7:**
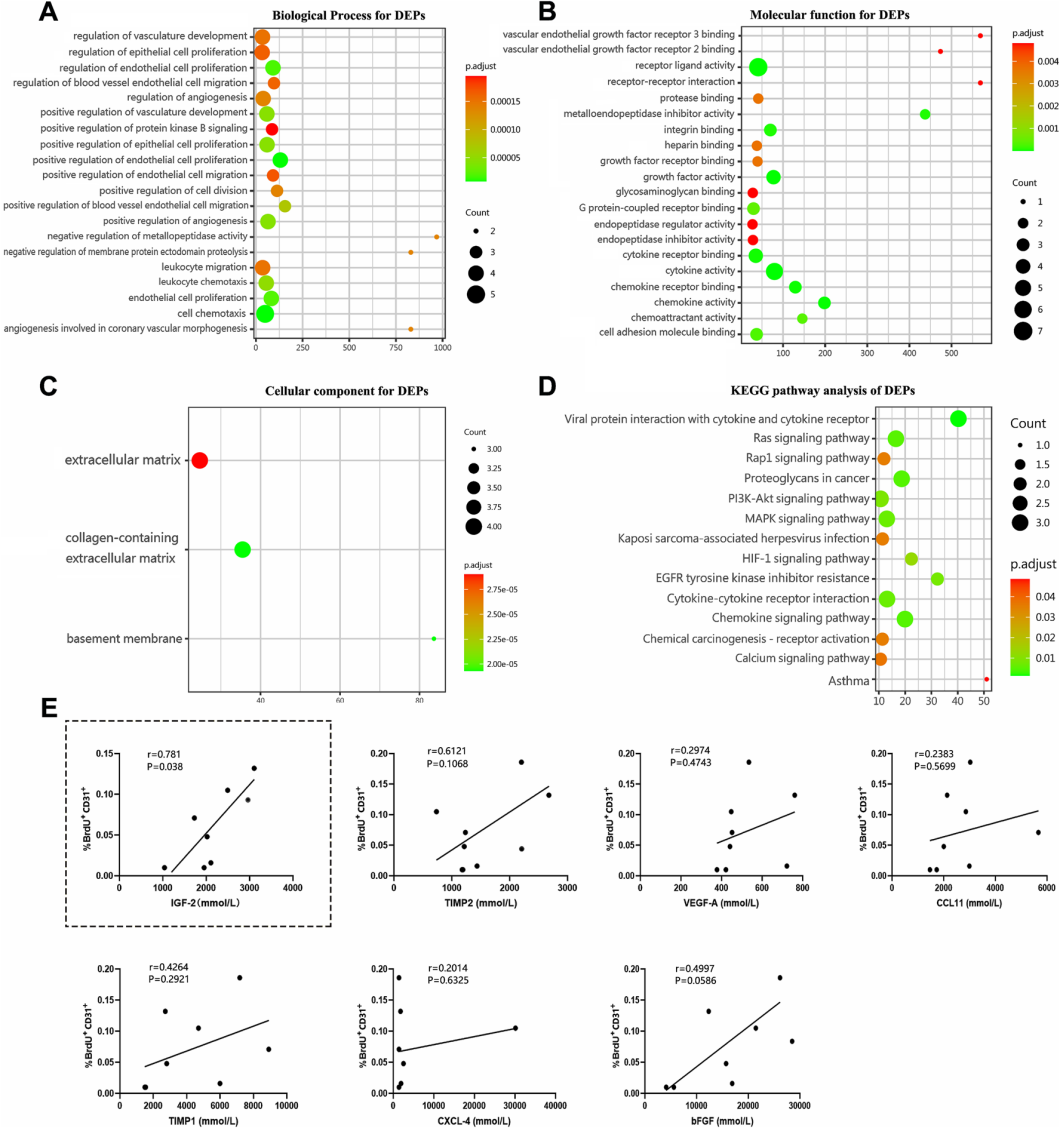
GO enrichment map and KEGG pathway enrichment map of differentially expressed proteins in mice. **A** The top 20 BP terms in the enrichment analysis. **B** The top 20 MF terms in the enrichment analysis. **C** The top 20 CC terms in the enrichment analysis. **D** The top 14 KEGG pathway terms of DEPs. **E** Pearson correlation of DEPs level and number of BrdU+/CD31 + cells on day 14 post-injury

To gain insight into the biological functions and signaling pathways linked with these seven proteins, we performed GO annotation and KEGG pathway analysis. The top 20 enriched items based on the P-value are shown in Fig. 7. Within the BP category, the most enriched items included positive regulation of endothelial cell proliferation, cell chemotaxis, and endothelial cell proliferation (Fig. 7A). The MF category showed significant enrichment for receptor ligand activity, cytokine activity, and growth factor activity (Fig. 7B). The CC terms were predominantly associated with collagen-containing extracellular matrix, basement membrane, and extracellular matrix (Fig. 7C). The 14 most significantly enriched KEGG pathway included viral protein interaction with cytokine and cytokine receptor, chemokine signaling pathway, Ras signaling pathway and PI3K-AKT signaling pathway (Fig. 7D).

To ascertain whether the seven DEPs were related to angiogenesis in ischemic brain tissue in this study, we carried out a Pearson correlation analysis between the seven DEPs and the percentage of BrdU^+^/CD31^+^ cells in the peri-infarct area (Fig. 7E). Remarkably, the analysis revealed a strong positive correlation between the levels of IGF-2 in ischemic brain tissue and angiogenesis (r = 0.781, P = 0.038). These findings suggest that the compounds may promote angiogenesis in ischemic brain tissue by upregulating the expression of IGF-2.

## Molecular docking analysis

**Fig. 8:**
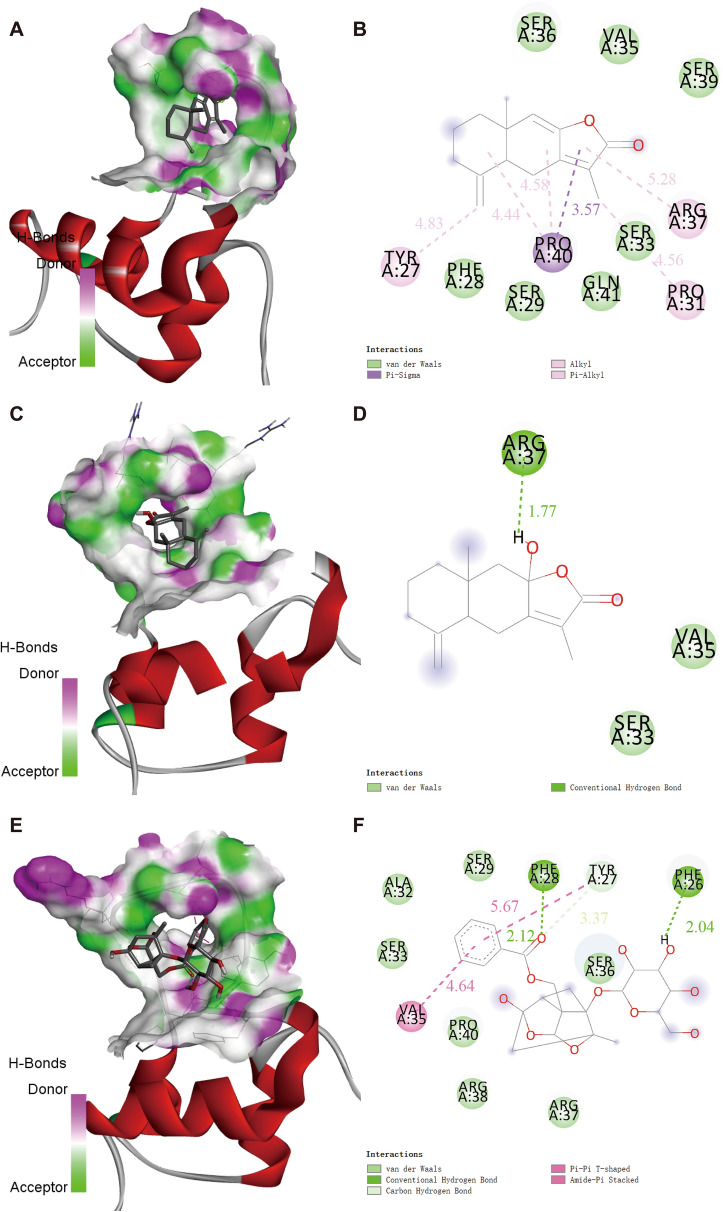
Molecular docking analysis of Atr I, Atr III and Pae with IGF-2 protein. **A**, **C** and **E** Three-dimensional interaction mode between Atr I Atr III and Pae with IGF-2, respectively. The potential binding sites show Atr I, Atr III, and Pae (in red) interacting with IGF-2 (in green). **B**, **D** and **F** Two-dimensional interaction mode between Atr I, Atr III and Pae with IGF-2

To investigate the possible interaction of Atr I, Atr III and Pae with IGF-2, a docking analysis was performed (Fig. 8A–I). The binding energies of Atr I, Atr III and Pae with IGF-2 protein were − 25.37 kJ/mol, − 21.77 kJ/mol and − 15.11 kJ /mol, respectively. While these values indicate some level of affinity between the compounds and IGF-2 [41]. The 3D view (Fig. 8A, C and E) clearly illustrate the types of interactions and the distances between the compounds and the target protein. The 2D views (Fig. 8B, D and F) reveal the primary binding sites for molecular docking, which include Leu32, Cys70, Cys75, Leu77 and Leu80 in Atr I; Cys75 in Atr III, Ser46 and Phe50 in Pae. These interactions contributed to the hypothesized binding pattern of Atr I, Atr III and Pae with IGF-2.

### Compounds regulates IGF-2/Ras/Raf/ERK and IGF-2/PI3K/AKT signaling in the MCAO mice model

**Fig. 9:**
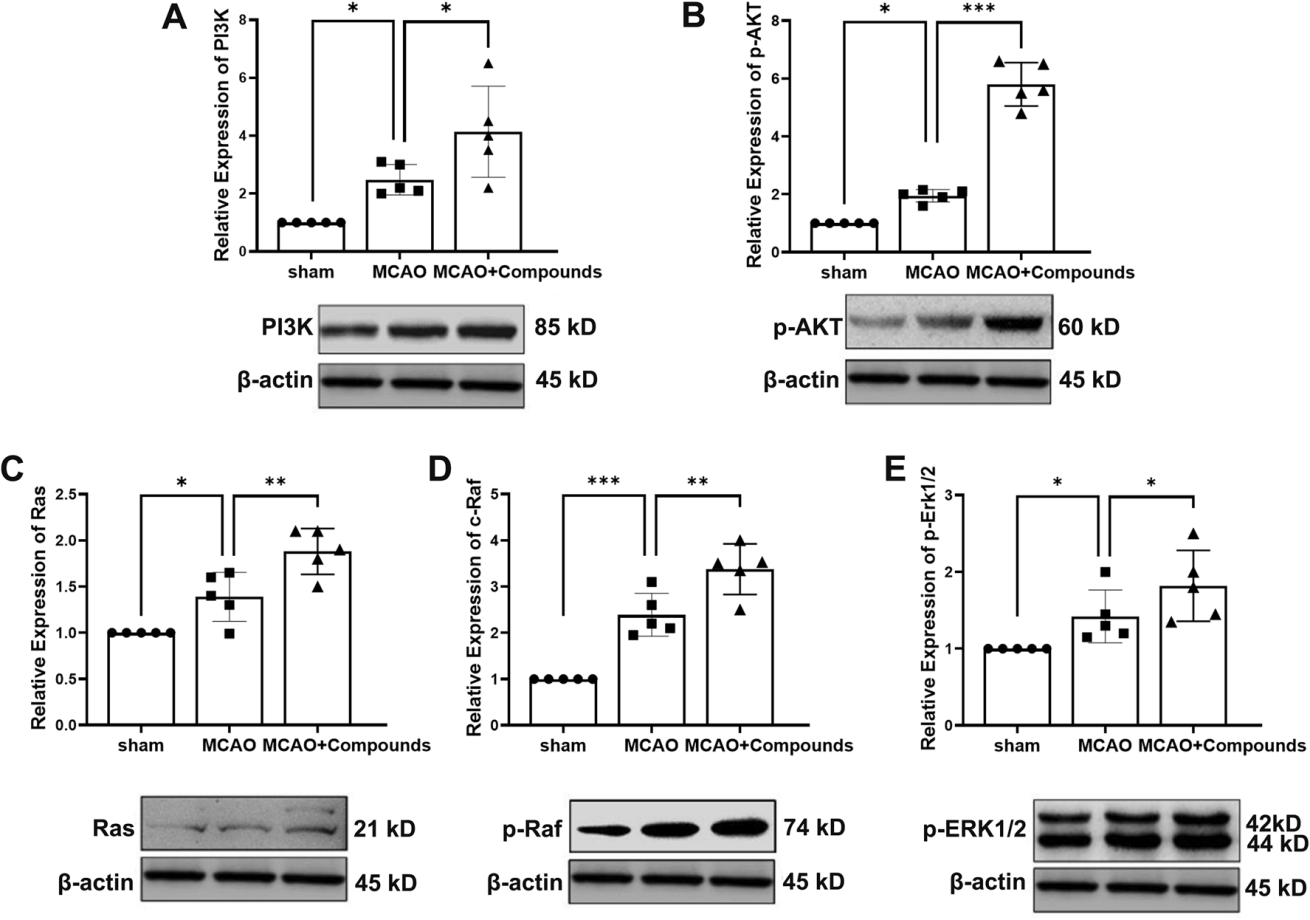
Effect of compounds on IGF-2/ Ras/ Raf/ ERK and IGF-2/ PI3K/ AKT signaling pathways. Western blot analysis was conducted on infarct hemisphere brain tissue at day 7 post-MCAO. The Western blots show levels of PI3K (**A**), p-AKT (**B**), Ras (**C**), p-Raf (**D**) and p-ERK1/2 (**E**). *n* = 5/group. *P < 0.05, **P < 0.01, ***P < 0.001

**Fig. 10:**
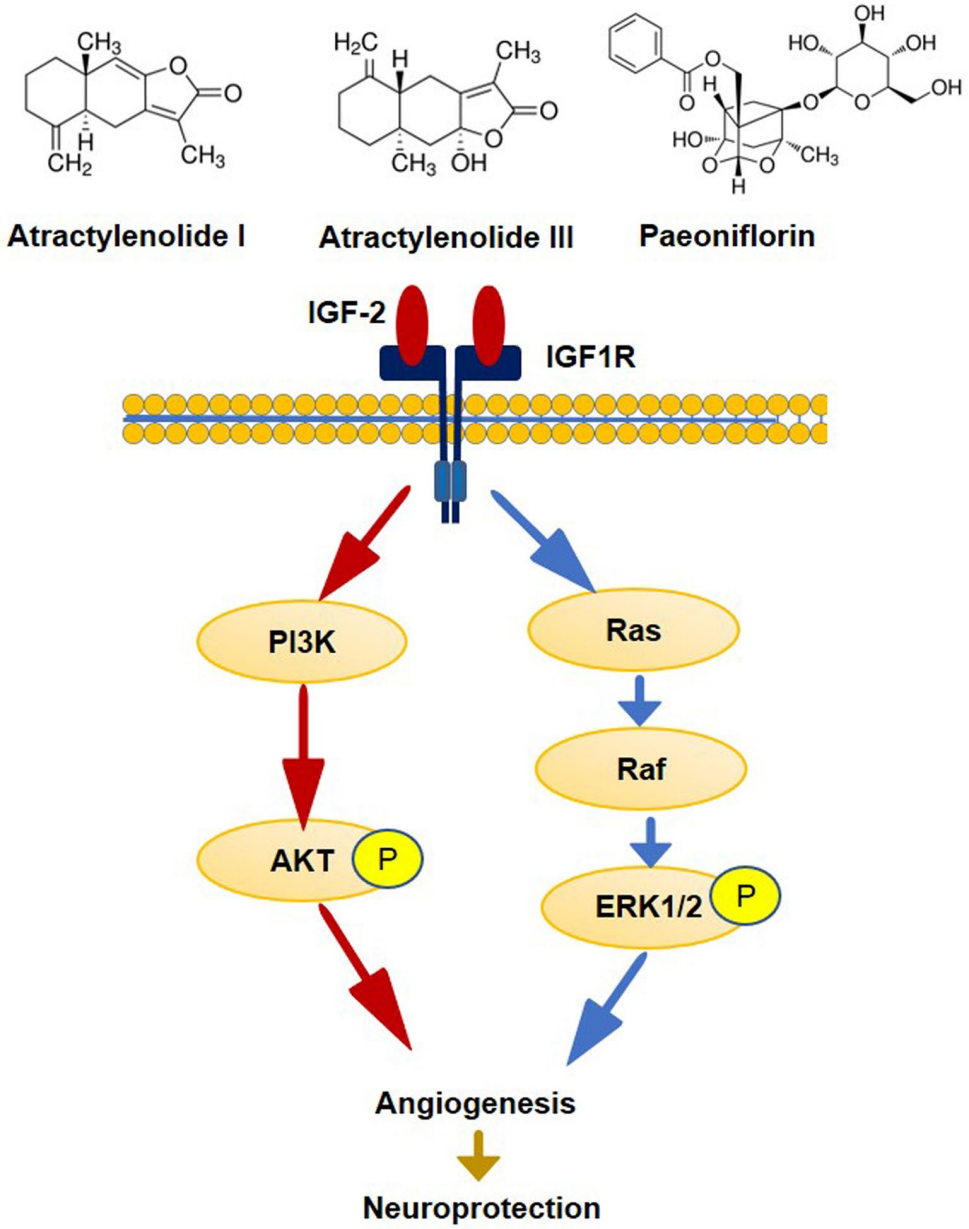
Proposed molecular mechanism of the compounds promoting angiogenesis in a mouse model of ischemic stroke

Previous studies have shown that IGF binding to the IGF-1 receptor activates multiple signaling pathways, including the Ras/ Raf/ ERK pathway and the PI3K/ Akt pathway [42]. Combining with the above KEGG analysis and Angiogenesis Antibody Array results, we detected the expressions of the pathway molecules. In agreement with above results, compounds significantly activated the PI3K and p-AKT in the ischemic hemisphere tissue (MCAO + Compounds vs. MCAO, P < 0.05, P < 0.0001, respectively) (Fig. 9A and B), The effect of compounds on Ras, p-Raf and ERK activity was also confirmed in the ischemic hemisphere tissue (MCAO + Compounds vs. MCAO, P < 0.01, P < 0.01 and P < 0.05, respectively (Fig. 9C-E). Our findings provide insight into the molecular mechanism of compounds as a therapeutic agent for promoting angiogenesis after ischemic stroke by activating IGF-2/ Ras/ Raf /ERK and IGF-2/ PI3K/ AKT pathway (Fig. 10).

## Discussion

The present study aimed to investigate the potential of AMK and PLP extracts in promoting angiogenesis and improving neurological recovery post-ischemic stroke. Our data revealed that the combination of Atr I, Atr III, and Pae significantly improved neurological recovery by stimulating angiogenesis following cerebral ischemia. In our in vitro experiments, we observed that these compounds facilitated angiogenesis by promoting endothelial cell proliferation, migration, and tube formation. These results were further supported by our in vivo study. Further investigation indicated that the compounds markedly influenced the expression of pivotal angiogenic proteins, such as IGF-2, TIMP-2, VEGFA, CXCL-4, and bFGF, while concurrently reducing levels of CCL11 and TIMP-1 in ischemic brain tissue. Notably, the upsurge in IGF-2 expression appeared to be intimately associated with augmented angiogenesis, suggesting that the compounds may promote angiogenesis primarily through the upregulation of IGF-2 and associated proteins.

The formation of new blood vessels post-ischemic stroke is crucial for the recovery of damaged tissue [43, 44]. It facilitates the delivery of oxygen and nutrients to the affected area, promoting repair and regeneration. Studies have consistently shown that enhancing angiogenesis can improve the prognosis of ischemic stroke patients by reducing tissue damage and improving functional outcomes [5, 9].

Building on this foundation, our study aimed to investigate the potential of multi-drug combinations in enhancing the angiogenic process. While our results suggest that the three-drug combination did not lead to a statistically significant increase in cell proliferation, migration and tube formation compared to the two-drug combinations. Given this complexity, we propose that the three-drug combination might offer a more holistic therapeutic approach. Although individual two-drug combinations may target one or two aspects of the angiogenic process effectively, the integration of all three drugs could potentially support a more robust and functional angiogenic response. It is this potential for a comprehensive effect that leads us to posit that the triple-drug combination could exert a more substantial impact on the promotion of angiogenesis, thereby offering significant therapeutic advantages.

In light of these considerations, we have chosen to proceed with the three-drug combination for our in vivo studies. This decision is informed by the possibility that a multi-faceted approach to angiogenesis may be more beneficial for the repair and regeneration of ischemic stroke-affected tissues. Our findings thus provide valuable insights into the potential therapeutic applications of TCM in the context of ischemic stroke treatment, suggesting that a combination of multiple compounds may be key to maximizing therapeutic efficacy.

Our findings suggest that extracts of AMK and PLP could enhance angiogenesis, highlighting their potential role in regulating “blood”. Moreover, the improvement in neurological function and recovery observed with the treatment of these compounds may also hint at their capability to replenish “Qi”. We speculate that this could be due to the increased cerebral blood flow from angiogenesis, resulting in improved delivery of nutrients and oxygen. This enhancement supports the brain’s metabolic needs and aids in the reestablishment of normal physiological functions [9] .

Research indicates that following cerebral ischemia and reperfusion in mice, there is a rapid expression increase in angiogenesis-related genes, with over 40% showing heightened activity within just an hour. These changes are not fleeting; they remain significant both 1 and 21 days after the ischemic event [45]. In human stroke patients, a similar trend is observed, with elevated levels of angiogenic proteins. Additionally, postmortem examinations have revealed a greater microvessel density in the vicinity of the infarcted tissue [46]. These findings collectively suggest that angiogenesis is not merely a passive response but an active contributor to the body’s recovery from cerebral ischemia.

Through protein microarray and bioinformatics analysis, we discovered that treatment with the compound combination led to the upregulation of key proteins involved in angiogenesis, including IGF-2, TIMP-2, VEGFA, CXCL-4, and bFGF, and to the downregulation of CCL11 and TIMP-1. These proteins are pivotal in promoting endothelial cell proliferation and migration, which are critical processes for the sprouting of new blood vessels [9, 47]. For example, VEGF-A not only stimulates endothelial cell proliferation and enhances migration but also inhibits apoptosis and increases vascular permeability [48]. IGF-2 is crucial for maintaining the phenotype of tip cells, which guide the sprouting of new blood vessels [42].

The differential expression of IGF-2, TIMP-2, VEGFA, and bFGF observed in our study highlights the complex nature of the biological response to cerebral ischemia and the potential influence of therapeutic interventions. The upregulation of these proteins in the MACO group is consistent with the endogenous reparative response following ischemic injury, emphasizing their roles in neuroprotection and tissue recovery processes such as angiogenesis and matrix remodeling. Interestingly, the drug treatment appears to further promote the expression of these proteins, which might suggest a synergistic effect with the body’s own healing mechanisms, possibly contributing to improved recovery outcomes.

While the study focused on these key proteins due to their established roles in stroke recovery, other proteins that showed altered regulation across different groups, such as IMP-1 and CCL11, were also noted. Despite their changes in expression, the absence of a direct link to angiogenesis in our analysis led to their exclusion from the primary discussion. Nonetheless, these proteins may still play significant roles in the broader context of stroke recovery and warrant further investigation. The insights gained from such analysis are crucial for a more comprehensive understanding of the molecular underpinnings of stroke recovery and the development of more effective therapeutic strategies.

Bioinformatics analysis further revealed that these differentially expressed proteins are primarily involved in biological processes critical to angiogenesis. They are also associated with well-established signaling pathways that govern cellular proliferation, growth, and migration. Among the pathways identified, the Ras signaling pathway stood out as a pivotal conduit, especially the Ras/Raf/MEK/ERK cascade, which is recognized for its crucial role in the formation of new blood vessels [49].Our findings suggest that the pro-angiogenic effects of the compounds could be mediated via the IGF-2 signaling axis. Elevated levels of IGF-2 were associated with enhanced endothelial cell proliferation and vessel formation, indicating a possible mechanistic connection. Furthermore, molecular docking analysis supports the likelihood of a direct interaction between the compounds and IGF-2. The response of IGF-2 to these compounds is noteworthy, given that IGF-2 is a powerful regulator of angiogenesis. It is known to boost the expression of vascular endothelial growth factor (VEGF) and simultaneously suppress anti-angiogenic factors [50].

In this study, we investigated the regulatory effects of compounds on the IGF-2/ Ras/p-Raf/ ERK and IGF2/ PI3K/ AKT signaling pathways in a mouse model of MCAO. Previous studies have established that IGF binding to the IGF1 receptor activates these signaling pathways, which are crucial for angiogenesis [42]. Our findings are consistent with this, as demonstrated by the significant activation of both the PI3K/Akt and Ras/p-Raf/ERK pathways in the ischemic hemisphere of mice treated with these compounds. Supported by our KEGG analysis and Angiogenesis Antibody Array data, these results suggest that the compounds may promote angiogenesis by stimulating these signaling cascades.

While this study offers valuable insights into the angiogenic properties of the TCM compounds under investigation and their potential therapeutic applications for ischemic stroke, it is important to recognize certain limitations. The experimental design could be improved by including positive and negative control groups in the animal model experiments to confirm the compounds’ efficacy and specificity. Such controls would allow for a more thorough evaluation of the compounds’ effects and strengthen the validity of our conclusions. Furthermore, to ensure the relevance of our findings for human health, the translational potential should be assessed through clinical trials aimed at verifying the compounds’ effectiveness in aiding stroke recovery.

## Data Availability

Yes.

## Abbreviations

Atr I: Atractylenolide I
Atr III: Atractylenolide III
Pae: Paeoniflorin
AMK: *Atractylodes macrocephala *Koidz
PLP: *Paeonia lactiflora* Pallas
MCAO: Transient middle cerebral artery occlusion
IGF-2: Insulin-like growth factor 2
TIMP-2: Metalloproteinase inhibitor 2
VEGFA: Vascular endothelial growth factor A
CXCL-4: C-X-C motif chemokine 4
bFGF: Fibroblast growth factor
VEGF: Vascular Endothelial Growth Factor
CCL11: C-C motif chemokine 11
TIMP-1: Metalloproteinase inhibitor 1
TCM: Traditional Chinese Medicine
OGD: Oxygen–Glucose Deprivation
DEPs: Differentially expressed proteins
GO: Gene Ontology
BP: Biological process
MF: Molecular function
CC: Cellular component
KEGG: Kyoto Encyclopedia of Genes and Genomes
PI3K: Phosphatidylinositol-3-hydroxykinase
p-AKT: Phosphorylated protein kinase B
Ras: Rat sarcoma
p-Raf: Phosphorylated rapidly accelerated fibrosarcoma
ERK: Extracellular signal-regulated kinase
